# The effect of transdermal estradiol or oral conjugated oestrogen and fenretinide versus placebo on haemostasis and cardiovascular risk biomarkers in a randomized breast cancer chemoprevention trial

**DOI:** 10.3332/ecancer.2008.67

**Published:** 2008-02-06

**Authors:** M Lazzeroni, D Macis, A Decensi, S Gandini, MT Sandri, D Serrano, A Guerrieri-Gonzaga, H Johansson, S Mora, C Daldoss, U Omodei, B Bonanni

**Affiliations:** 1 Cancer Prevention and Genetics, European Institute of Oncology, 20141 Milan, Italy; 2 Epidemiology and Biostatistics, European Institute of Oncology, 20141 Milan, Italy; 3 Laboratory Medicine, European Institute of Oncology, 20141 Milan, Italy; 4 University Clinics Obstetrics and Gynecology, Brescia, Italy; 5 Division of Medical and Preventive Oncology, E.O. Ospedali Galliera, Genoa, Italy

## Abstract

**Background:**

We have previously reported the favourable effect of transdermal estradiol (E2), relative to oral conjugated equine oestrogen (CEE), on ultrasensitive C-reactive protein after 12 months of treatment in a retinoid-placebo controlled two-by-two randomized breast cancer prevention trial (Decensi A *et al* (2002) *Circulation* **106** 10 1224–8). Here, we investigate the changes in lipids and clotting profile in patients of the same trial.

**Methods and results:**

Recent post-menopausal women were randomised to either oral CEE 0.625 mg/day and placebo (*n* = 55), CEE and fenretinide 200 mg/day (*n* = 56), transdermal E2 50 mg/day and placebo (*n* = 59) or E2 and fenretinide 200 mg/day (*n* = 56). Sequential medroxyprogesterone acetate 10 mg/day was given in each group. After 12 months, there was a statistically significant effect of the route of administration of hormone replacement therapy (HRT) on fibrinogen levels; the median percentage change being −5.7% with CEE and −1.1% with E2 (p = 0.012). Total cholesterol decreased in all arms (p < 0.0001). HDL-C decreased significantly with transdermal E2 (p = 0.006) compared to oral CEE and with fenretinide relative to placebo (p<0.001). Triglycerides exhibited an opposite modulation in the HRT route, with a 21.4% median increase with oral CEE and an 8.6% reduction with transdermal E2 (p < 0.0001). Antithrombin-III showed a 4% borderline significant reduction in the fenretinide arm relative to placebo, irrespective of the HRT administration route (p = 0.055).

**Conclusions:**

Our data indicate that transdermal E2 may be preferable to oral CEE based on its safer cardiovascular risk profile. Fenretinide modified some cardiovascular risk biomarkers and confirmed a safer profile compared to other retinoids.

## Background

Coronary heart disease (CHD) is the leading cause of death and disability in women in industrialized countries [1]. Pre-menopausal women rarely suffer from cardiovascular diseases, while the menopause, regardless of age at onset, is associated with a marked increase in CHD risk [1,2]. Some observational studies showed that the biological effects of hormone replacement therapy (HRT)—i.e. a favourable modulation of lipid profile tone of vascular smooth muscle cells and fibrinogen levels—may reduce cardiovascular risk by about 50% [3,4].

However, evidence from recent clinical trials has challenged these notions [5,6]. The HERS trial (Heart and Oestrogen/Progestin Replacement Study) in women previously affected with CHD showed no overall benefit from HRT, but a significant excess of venous thromboembolic events and a higher incidence of cardiovascular episodes in the oral conjugated equine oestrogen (CEE)/ medroxyprogesterone acetate (MPA) group compared with the placebo group in the first year [5]. To explain such findings, research into the effects of HRT on haemostasis/coagulation activation and a pro-inflammatory action was advocated [5].

The Women’s Health Initiative (WHI), a large primary prevention trial, reported more myocardial infarctions and venous thromboembolic events after treatment, with oral CEE and MPA, confirming a similar pattern of events as the HERS [6].

Previous studies examined the effect of HRT on the levels of plasminogen activator inhibitor-1 (PAI-1), a biomarker able to inhibit both the tissue plasminogen activator and urokinase plasminogen activator; thus blocking the activation of plasminogen to plasmin [7,8]. So elevated levels of PAI-1 inhibit fibrinolytic activity and may be a risk factor for acute coronary events [9]. Some studies reported a significant decrease in PAI-1 plasma levels with HRT, which was more pronounced with oral compared to transdermal HRT [10,11].

Fenretinide [*N*-(4-hydroxyphenyl) retinamide (4-HPR)] is a synthetic amide derivative of retinoic acid, which has been shown to exhibit significant anti-tumour activity in both oestrogen receptor (ER)-positive and ER-negative breast cancer cells and to decrease *bcl-2* mRNA levels with a consequent pro-apoptotic effect. Although the specific mechanisms of action of fenretinide are not fully elucidated, this apoptotic effect is mediated by increased ceramide production and accumulation of reactive oxygen species [12–14].

In a phase III trial with nearly 3000 women with early breast cancer, fenretinide decreased by 38% the risk of a second breast malignancy in pre-menopausal women but not in post-menopausal women. Notably, this protective effect lasts as long as ten years after treatment cessation [15].

Based on the potential sensitizing effect of sex steroids underlying the preventive effect of fenretinide in pre-menopausal women, we conducted a phase IIb trial in 226 recent post-menopausal healthy women with menopausal symptoms who were randomized to receive either oral CEE, transdermal E2 and fenretinide or placebo. The trial was designed to investigate the effect of fenretinide and oral CEE, or transdermal E2, at biologically comparable doses on biomarkers of breast cancer and cardiovascular risk in healthy women undergoing HRT [16]. We observed no change of ultrasensitive C-reactive protein (CRP) with transdermal E2 compared with an increase with oral CEE. This may explain the lower CHD risk associated with transdermal E2 [16]. In addition, we found that transdermal E2 has different effects to oral CEE on circulating IGF-I and SHBG, but is associated with a similar increase in mammographic density [17].

To provide insight into the effects of fenretinide and the different routes of HRT administration on biomarkers of CHD risk, we investigated in a randomized trial the changes in lipids and clotting profiles, including total cholesterol high-density lipoprotein cholesterol (HDL-C), low-density lipoprotein cholesterol (LDL-C), triglycerides antithrombin III (AT-III), fibrinogen platelets and PAI-1, after 12 months of transdermal E2, oral CEE and fenretinide or placebo.

## Methods

### Subjects

The main subject characteristics have been detailed in Table 1. Study participants were post-menopausal healthy women *de novo* HRT users with 6–60 months amenorrhoea and follicle-stimulating hormone levels >40 U/L, who were willing to receive HRT for menopausal symptom relief. Four centres in Italy were involved in the study: the European Institute of Oncology, Milan and the University Clinics of Obstetrics and Gynaecology of Brescia, Varese and Trieste.

### Assignment

The study is a randomized double-blind placebo controlled phase IIb trial. Eligible women were randomized to receive either transdermal E2 (50 mg/day) released by a weekly patch (Climara Schering SpA) and fenretinide (RW Johnson Pharmaceutical Research Institute) two oral capsules (100 mg each) administered daily during dinner (*n* = 56) or transdermal E2 and oral placebo capsules (*n* = 59), 0.625 mg/day of oral conjugated oestrogens (Premarin Wyeth-Lederle) and fenretinide (*n* = 56) or oral CEE and oral placebo (*n* = 55) for one year. Sequential medroxyprogesterone acetate (MPA Farlutal Pharmacia) at 10 mg/day p.o. for the first 12 days of each month was added to continuous oestrogen therapy in each arm. A three-day rest period from the capsules (fenretinide or placebo) was prescribed at the end of each month in order to allow a sufficient retinol uptake for correct night vision [15].

Randomization was centrally performed by telephone at the European Institute of Oncology, using permuted blocks of four and stratified by centre and route of HRT.

### Laboratory methods

At baseline and at a 12-month visit, fasting blood samples were taken between 8 and 10 a.m. Samples were appropriately centrifuged and aliquoted for fresh and frozen measurements.

Platelet count was performed with an automatic instrument (SE 9000 Sysmex, Japan).

Total cholesterol, HDL-C triglycerides and antithrombin-III levels were measured in the serum by enzymatic methods with a Hitachi 911 (Boehringer-Mannheim Germany), a fully mechanized multi-channel analyser for routine clinical chemistry purposes. Measurements were taken according to the manufacturer’s instructions. Total cholesterol HDL-C and triglyceride determinations were based on enzymatic colorimetric methods, whereas antithrombin III levels were determined by a kinetic colorimetric method. LDL-C levels were obtained according to the Friedewald formula (LDL-C = total cholesterol – HDL-C – [triglycerides/5]) [18]. Plasma fibrinogen was measured using the ELECTRA 1400C Automatic Coagulation Analyzer (Medical Laboratory Automation Inc., USA). The assay method is based on the photometric determination of blood clot formation.

PAI-1 was determined by an ELISA sandwich technique (Asserachrom PAI-1 Diagnostica Stago France). The intra-assay and inter-assay coefficients of variations were 5.48% and 8.69%, respectively.

Lipid profiles, platelet number, fibrinogen and antithrombin-III levels were determined on fresh specimens, while PAI-1 was measured in pre- and post-treatment frozen serum samples, obtained from each subject within the same work session, to reduce the effects of inter-assay variation.

### Statistical methods

The values of biomarkers at baseline and 12 months were analysed using repeated measures models, taking into account the correlation within subjects with a compound symmetry covariance matrix. Time was treated as a factor and the effects of time on HRT route and fenretinide treatment groups were included in the model. All interactions were tested using F-tests based upon Type 3 sums on squares. These analyses relied on a normal distribution of biomarkers. Such an assumption was graphically checked on residuals from saturated models. Log transformations were used when necessary.

Analyses with mixed-effect models were performed using PROC MIXED with the SAS Software^®^ (SAS Institute, Inc., Cary, NC). Two-sided p values below the 5% threshold were regarded as statistically significant.

## Results

Median and inter-quartile ranges of haemostatic biomarkers and lipids at baseline, and at 12 months, are reported in Table 2—together with the median percentage changes from baseline.

After one year of treatment, all the biomarkers, with the exception of triglycerides, varied compared to baseline.

**Figure 1:**
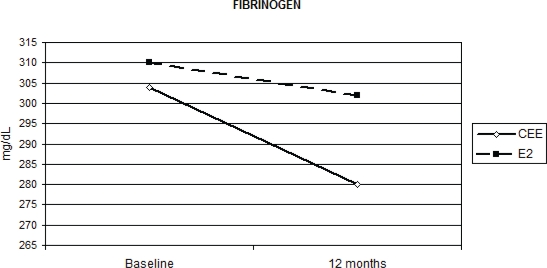
Effect of HRT on plasma fibrinogen concentration at baseline and after 12 months of treatment (p = 0.012)

HRT significantly reduced fibrinogen AT-III and PAI-1 without significant differences between the two routes of administration, except for fibrinogen (Table 2). Relative to baseline fibrinogen decreased by 5.7%, with oral CEE, versus 1.1% with transdermal E2 (p = 0.012 for the interaction Figure 1) after 12 months. Compared with the placebo, fenretinide had no effect on the changes in haemostatic biomarkers, but a trend to a reduction in AT-III (median percentage change = −4% versus 0% on placebo p = 0.055 for the interaction, as shown in Table 2).

When compared with baseline, both routes of HRT significantly reduced LDL-C and total cholesterol. A significant difference between routes for HDL-C and triglycerides was found (Table 2).

**Figure 2:**
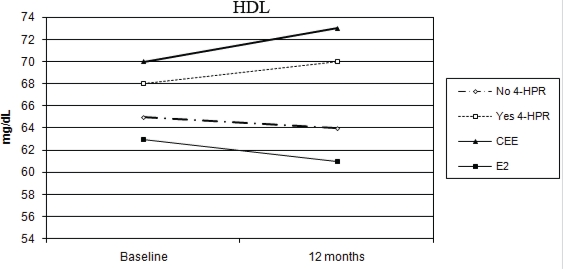
Different modulation of HDL-C in the four arms of treatment (CEE versus E2: p < 0.006, without 4-HPR versus with 4-HPR: p < 0.001)

No percentage change of HDL-C was observed in the oral CEE group compared with a 4% decline in the transdermal E2 group (p = 0.006 for the interaction between time and HRT route). In addition, a statistically significant difference was observed between fenretinide and placebo on HDL-C levels. Subjects on fenretinide had a median percentage increase of 1.4% after one year of treatment, in contrast to a decrease of 4.9% in the placebo group (p < 0.001 for the interaction between time and fenretinide). The different effects of HRT route and fenretinide versus placebo on the changes in HDL-C after 12 months are illustrated in Figure 2.

**Figure 3:**
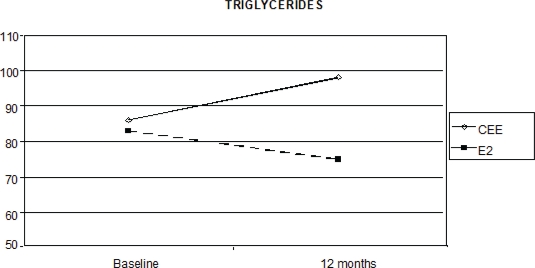
Effect of HRT on triglyceride concentration at baseline and after 12 months of treatment (p < 0.0001)

Triglycerides levels also showed a statistically significant interaction between time and the HRT route, as the median percentage increase was 21.4% with oral CEE, versus a reduction of 8.6% with transdermal E2 (p < 0.0001 Figure 3).

There was no difference in any biomarker change according to first-degree family history of cardiovascular disease (data not shown).

## Discussion

The choice of the most appropriate form of HRT is an important clinical issue in women with menopausal symptoms.

Previous pilot studies have shown that transdermal E2 and oral CEE have a remarkably different pattern of hormonal metabolic and vascular effects presumably as a consequence of the high concentration of orally administered oestrogens at first-pass hepatic level [16,19,21]. In addition, it has been shown that transdermal E2 in contrast to the oral CEE does not increase CRP levels in healthy women, further supporting the notion that transdermal E2 and oral CEE exert different effects on CHD risk factors. Also, the effect of HRT on coagulation activation is controversial [5,11,22,25]. The results of our large randomized biomarker trial indicate that all the haemostatic biomarkers we analysed in subjects treated with HRT significantly decreased, irrelevant of the route of HRT administration. A significantly different modulation between oral and transdermal administration was shown only for fibrinogen and triglycerides. It has been estimated that increased plasma fibrinogen level reflects an inflammatory state of the vascular wall, which is among the main mechanisms of atherosclerosis [26]. Some studies showed a significant decrease in fibrinogen with both routes of HRT—although more pronounced with oral CEE [25–27]—while others found no significant difference [21–28]. This divergence might be due to design characteristics, patient number, treatment, regimens, type of HRT used, duration of treatment and follow-up, but also genetic polymorphisms [29]. In our study, relative to baseline, fibrinogen had a greater decrease after 12 months of oral CEE than transdermal E2, possibly as a result of higher plasma levels of oestrogens obtained with the oral administration. Our data add further support to the notion that HRT could have a protective effect on this independent risk factor for ischemic heart disease.

In contrast to oral CEE, transdermal E2 has been shown to have only minor favourable effects on serum, lipids, lipoproteins and fibrinolysis [10–30]. A recent study showed how different hormone therapy regimens may exhibit a different effect on lipid profile. Specifically, CEE+MPA had no effect on HDL-C, probably as a consequence of an antagonist effect of MPA on the oestrogen induced increase of HDL-C [31]. Our data confirm this hypothesis as they show a different effect of transdermal E2 versus oral CEE. We observed a similar decrease in total cholesterol and LDL-C in both routes. HDL-C showed a statistically significant decrease after transdermal E2 and no change in the oral group. HDL-C showed an increasing trend in fenretinide users, versus a decreasing trend in the placebo group. If we assume that the decrease in HDL-C is probably due to increased catabolism (MPA could have generated a net progestogenic/androgenic hepatic effect via hepatic lipase to decrease HDL-C; thus neutralizing the effect of E2 [32]), rather than a decrease in synthesis, the pathological significance may be minimal.

The complexity of the lipid–lipoprotein factor and the difficulty in obtaining a clear evaluation on the net effects of this factor on the cardiovascular system becomes evident when considering triglycerides. The importance of serum triglycerides as a risk factor for cardiovascular diseases is controversial, and many studies [33,34] have different results, probably due to regression dilution bias [35] or differences in the triglyceride measurement system adopted [36]. Two meta-analyses [36,37] provided robust evidence that serum triglyceride levels are associated with the risk of developing cardiovascular disease independent of other major measured risk factors including HDL-C. For this reason, a reduction in triglycerides is favourable because increased triglycerides concentrations (particularly the large triglyceride-rich fraction) are independently linked to CHD risk, particularly in women [38], and they can promote endothelial damage oxidation and inflammatory changes in the vascular endothelium [39].

In our study, CEE+MPA was associated with an increase in triglycerides. Previous studies confirmed this trend. Mercuro *et al* [40] and Lobo *et al* [41] reported similar magnitudes of triglycerides increase with CEE at 0.625 and 0.3 mg, whereas Sanada *et al* [42] and Wakatsuki *et al* [43] associated 0.3 mg with a less pronounced increase in triglyceride levels. Christodoulakos *et al* [31] pointed out that the increase of triglycerides was less evident in non-androgenic progestin arms, suggesting a difference in androgenic potency of the progestinic as causative factor. In our study, we saw an increase in triglycerides with oral CEE, but a reduction with transdermal E2. This trend is similar to a previous study [44], indicating that this difference may reflect the different pharmacokinetics of the two routes of administration.

Despite the limitations of this analysis, i.e. the absence of a blind arm for HRT, the different type of oestrogen for the two routes of HRT and the fact that this exploratory analysis is based on a study designed to investigate other endpoints, we present some new and interesting data about the effects of fenretinide on cardiovascular-risk biomarkers.

Several retinoids are known to increase triglyceride and LDL-cholesterol levels. In order to explain the increased cardiovascular mortality observed in the Carotene and Retinol Efficacy Trial (CARET), Cartmel *et al* suggested a possible contribution of hypercholesterolemia and hypertriglyceridemia in the treatment arm compared to placebo [45]. On the contrary, fenretinide has a neutral effect on tests of liver function, lipid profile and blood cell counts as compared to other retinoids [46]. Indeed, we found a favourable effect of fenretinide on HDL-C. Likewise Zujewski *et al* [47] reported a statistically significant increase of serum HDL-C in metastatic breast cancer patients treated with tamoxifen and fenretinide.

Interestingly, our data shows no effect of fenretinide on fibrinogen and triglyceride levels differently from other retinoids [45], but is consistent with our previous results [46,47]. The observed association between fenretinide administration and the reduction trend of AT-III is new and requires further investigation to assess its potential clinical impact.

## Conclusions

In conclusion, our data show a different profile of transdermal E2 and oral CEE on early CHD risk and provide new information about the effects of fenretinide on lipid and clotting profiles. If we consider the different balance between positive and negative effects of transdermal E2 and oral CEE on cardiovascular-risk biomarkers, both the more favourable effects on triglycerides by transdermal E2 and the neutral effect on CRP, support the notion that transdermal E2 may be associated with a safer profile when compared to oral CEE. We have confirmed the safer profile of fenretinide in comparison to other retinoids, but further studies are needed to better understand the possible role of this promising compound in chemoprevention.

## Figures and Tables

**Table 1:**
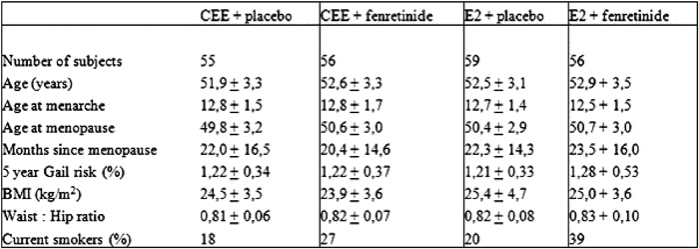
Main subject characteristics at baseline (mean ± SD)

**Table 2:**
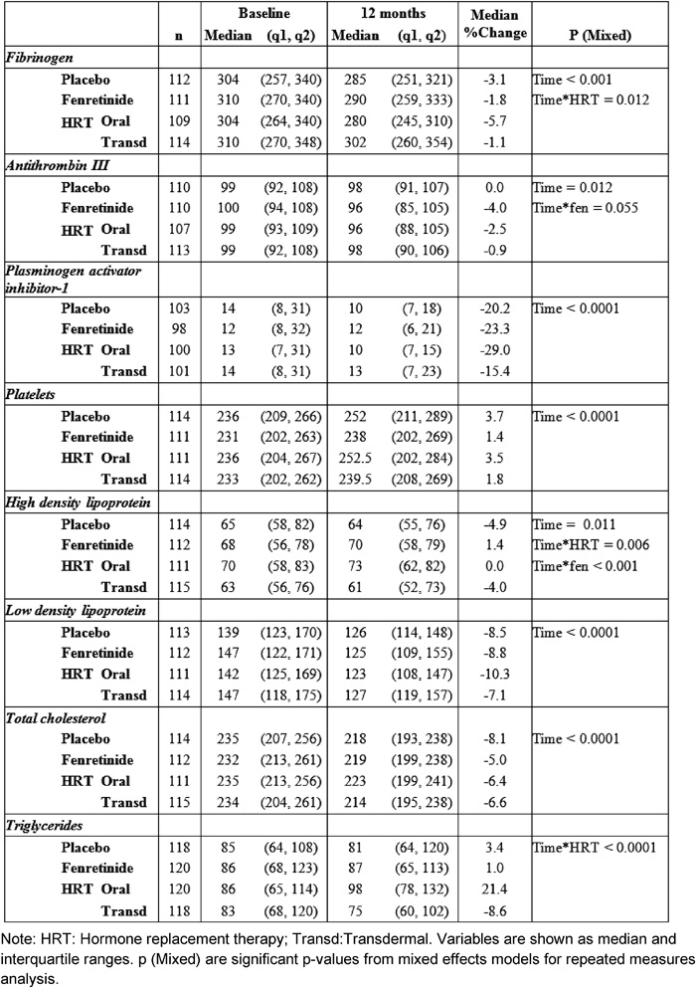
Circulating concentrations of haemostatic markers of and lipid profile at baseline and after 12 months of treatment
